# Attention Network with Information Distillation for Super-Resolution

**DOI:** 10.3390/e24091226

**Published:** 2022-09-01

**Authors:** Huaijuan Zang, Ying Zhao, Chao Niu, Haiyan Zhang, Shu Zhan

**Affiliations:** Key Laboratory of Knowledge Engineering with Big Data, Ministry of Education, School of Computer Science and Information Engineering, Hefei University of Technology, Hefei 230601, China

**Keywords:** image super-resolution, distillation structure, attention mechanism

## Abstract

Resolution is an intuitive assessment for the visual quality of images, which is limited by physical devices. Recently, image super-resolution (SR) models based on deep convolutional neural networks (CNNs) have made significant progress. However, most existing SR models require high computational costs with network depth, hindering practical application. In addition, these models treat intermediate features equally and rarely explore the discriminative capacity hidden in their abundant features. To tackle these issues, we propose an attention network with information distillation(AIDN) for efficient and accurate image super-resolution, which adaptively modulates the feature responses by modeling the interactions between channel dimension and spatial features. Specifically, gated channel transformation (GCT) is introduced to gather global contextual information among different channels to modulate intermediate high-level features. Moreover, a recalibrated attention module (RAM) is proposed to rescale these feature responses, and RAM concentrates the essential contents around spatial locations. Benefiting from the gated channel transformation and spatial information masks working jointly, our proposed AIDN can obtain a more powerful ability to identify information. It effectively improves computational efficiency while improving reconstruction accuracy. Comprehensive quantitative and qualitative evaluations demonstrate that our AIDN outperforms state-of-the-art models in terms of reconstruction performance and visual quality.

## 1. Introduction

The resolution of an image is restricted by the sensor imaging device, hindering its development. Single-image super-resolution (SISR) is a typical low-level problem in computer vision, which aims to restore an accurate high-resolution (HR) image from a degraded low-resolution (LR) observation. It has been widely used in various important fields involving the development of multimedia technology [1], such as remote-sensing imaging, live video [2], and monitoring devices. However, image super-resolution is still a challenging topic because multiple HR images may be reconstructed from any LR image. To tackle this difficulty, plenty of approaches based on deep convolutional neural networks (CNNs) have been proposed to establish LR–HR image mappings, which have achieved excellent performance [3,4].

SRCNN [5] was the pioneering work in deep learning for image super-resolution reconstruction, directly modeling an end-to-end mapping through only a three-layer convolutional network, which achieved better results than traditional algorithms. Subsequently, deep CNN-based SR models have become the mainstream. Kim et al. presented a very deep convolutional network (VDSR) [6] and DRCN [7], pushing the model depth to 20 layers by equipping the residual structure [8], which led to a remarkable performance gain (e.g., VDSR obtained a PSNR of 37.53 vs. SRCNN’s PSNR of 36.66 on Set5 ×2; PSNR is defined in Section 4.1). These methods take the interpolated LR image as the input to the network, undoubtedly increasing the computational burden and time overhead. FSRCNN [9], using transposed convolution, and ESPCN [10], adopting sub-pixel convolution, have been proposed to accelerate the inference and reduce the computational burden by changing the up-scaled position of the input low-resolution (LR) image. Thanks to effective sub-pixel convolution, Lim et al. [11] explored a broad and deep EDSR network without the batch normalization module, dramatically improving the SR perfromance (e.g., EDSR PSNR = 38.11 vs. SRCNN PSNR = 37.53 on Set5 ×2). Since then, researchers have attempted to design more complex networks to enhance network accuracy.

To obtain more abundant information, hierarchical features and multi-scale features can be used. Wang et al. [12] introduced an adaptive weighted multi-scale (AWMS) module residual structure to realize a lightweight network. SRDenseNet [13], based on DenseNet [14], used the concatenated features of all layers to enhance feature propagation and maintain continuous feature transmission. Furthermore, Song et al. [15] leveraged NAS [16] to find an efficient structure based on a residual dense module for accurate super-resolution. However, most SR models do not distinguish these intermediate features and lack flexibility in processing different information types, thus preventing better performance. RCAN [17] developed a channel attention module to model the channel interdependencies, in order to obtain discriminative information, and achieved a PSNR of 38.27 on Set5 ×2; however, it has more than 16M parameters, which is not conducive to deployment on resource-limited devices. Later, Hui et al. [18] constructed an information multi-distillation structure with the splitting operation, greatly reducing the number of channels. Lan et al. [19] introduced channel attention into the residual multi-scale module to enhance the feature representation capability (MADNet), and generated a PSNR of 37.85 on Set5 ×2 with 878 K parameters.

Motivated by the above, we propose an attention network with an information distillation structure (AIDN) for efficient SISR, using several stacked attention information distillation blocks (AIDB). Inspired by IDN, we carefully develop an attention information distillation block (AIDB) to asymptotically learn more intermediate feature representations, mainly employing multiple splitting operations combined with gate channel transformation (GCT). Specifically, the splitting strategy divides the previously extracted features into two parts, where one is retained while the other is further processed by GCT. The normalization method and attention mechanism are combined to gain precise contextual information. GCT can learn the importance of different channels adaptively and takes weighted feature maps as the input to the next layer. Meanwhile, GCT encourages cooperation at shallow layers and competition at deeper layers. Moreover, all distilled features are aggregated through the recalibrated attention module (RAM), which further refines these high-frequency features and revises the importance of features in the channel dimension. In general, the main contributions of our work can be summarized as follows:

- We propose an attention network with an information distillation structure (AIDN) for efficient and accurate image super-resolution, which extracts the valuable intermediate features step by step using the distillation structures;
- We introduce gate channel transformation (GCT) into SISR and use it in one distillation branch;
- We propose a recalibrated attention module (RAM) to re-highlight the contributions of features and strengthen the expressive ability of the network. Comprehensive experimental results demonstrate that the proposed method strikes a good balance between performance and model size.

## 2. Related Work

### 2.1. Deep CNN-Based Super-Resolution Methods

In recent years, methods based on deep convolutional neural networks (CNNs) have been successfully applied to various tasks, showing excellent performance.

Dong et al. [5] first explored the use of three convolutional layers for single-image super-resolution (SISR), and obtained better reconstruction results than by using the traditional methods. Subsequently, with the successful application of the residual network architecture [8] in computer vision tasks, more and more residual-learning-variant algorithms have been used to reconstruct SR images, including LapSRN [20], WMRN [21], CFSRCNN [22], and RFANet [23]. Dense connections have also been introduced for image super-resolution through the information flow of hierarchical features. RDN [24] combined the residual structure with dense connections to form a residual dense network with a continuous memory. Zhang et al. [25] developed GLADSR through the use of the global–local adjustment of dense connections to increase the network capacity.

Although these methods have achieved good performance, the parameters increase dramatically with the network depth, making them unsuitable for mobile platforms. DRCN [7] leveraged recursive learning to decrease the parameters of the network. CARN [26] developed a cascading architecture in the residual structure, forming a lightweight model suitable for practical applications. CBPN [27] struck a good balance between efficiency and performance by learning mixed residual features. Song et al. [28] devised AdderNets to resolve the defects of adder neural networks. It provided a better visual effect with lower energy consumption without changing the original structures. More recently, some NAS-based SR models have been proposed to automatically search for optimal architectures. Chu et al. [29] presented an automatic search algorithm, FALSR, based on NAS, to achieve a fast and lightweight SR model. DRSDN [30] explored diverse plug-and-play network architectures for efficient single-image super-resolution.

### 2.2. Attention Mechanism

Attention mechanism is a data processing method in machine learning, which is used to improve the performance of convolutional neural networks (CNN) in computer vision tasks. Attention mechanism aims to enable a network to automatically learn more focused areas by using masks (new weights). SENet [31] can be regarded as the first model of attention mechanism, which improved the representational capability of the network by modeling the relationship between channels. Wang et al. [32] presented a non-local block to calculate the response of a location to the information of all positions. CBAM [33] connected channel attention and spatial attention in a series to obtain a 3D attention map to form a lightweight, universal module. GCT [34] combined a normalization module with attention mechanism using lightweight variables to learn the interrelationships between channel-wise information. ECA-Net [35] developed a local cross-channel interaction scheme without dimension reduction, which proved to be an efficient and lightweight channel attention structure.

In addition, attention-based works have been proposed to further improve super-resolution performance. Zhang et al. [23] introduced enhanced spatial attention (ESA) into the residual-in-residual (RIR) structure to build a residual feature aggregation block, thus forming a lightweight and effective model. Dai et al. [36] designed a second-order attention network (SAN), which employed second-order feature statistics to learn more discriminative feature expressions. DRLN [37] developed a novel Laplacian attention with dense connections on the cascaded residual structure to study the inter- and intra-layer dependencies that achieved deep supervision. Hu et al. [38] explored channel-wise and spatial attention residual blocks (CSAR) to modulate hierarchical features in both global and local manners, achieving prominent performance. CSNLN [39] proposed a non-local attention with a different scale, which thoroughly explored all possible priors through non-local calculations of the feature-wise similarities between patches in cross-scales.

## 3. Proposed Method

### 3.1. Network Architecture

**Figure 1:**
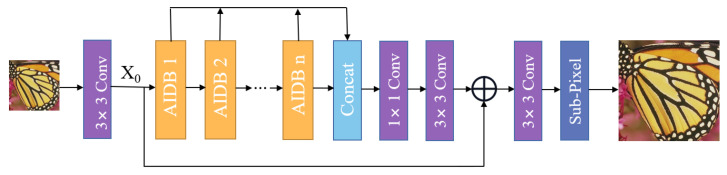
Overview of our AIDN architecture.

In this section, we introduce the entire framework of our proposed attention network with information distillation (AIDN), as shown in Figure 1. Our AIDN architecture comprises three parts: a low-level feature extraction module (LFE), stacked attention information distillation blocks (AIDBs), and a image reconstruction module. Here, $I_{LR}$ represents the original low-resolution (LR) input image, while $I_{SR}$ denotes its output super-resolution (SR) image. Specifically, a convolutional layer is first leveraged to extract the shallow features from the given LR input. This procedure can be expressed as

(1) $$X_{0}=F_{LFE}\left(I_{LR}\right)$$

where $F_{LFE}\left(\cdot \right)$ denotes a convolutional layer with a kernel size of 3×3, and $X_{0}$ is the extracted shallow features. Then, $X_{0}$ is sent to the next part, which consists of multiple attention information distillation blocks (AIDBs) in a chain, which gradually refines multiple hierarchical features. This process can be denoted as

(2) $$X_{n}=F_{AIDB}^{n}\left(X_{n-1}\right)=F_{AIDB}^{n}\left(F_{AIDB}^{n-1}\left(\cdots F_{AIDB}^{0}\left(X_{0}\right)\cdots \right)\right)$$

where $F_{AIDB}^{n}$ indicates the *n*-th AIDB function, and $X_{n-1}$ and $X_{n}$ denote the input and output feature maps of the *n*-th AIDB, respectively.

Then, the deep features generated by this sequence of AIDBs are further concatenated together through global feature fusion. After fusing, the deep features are processed by two convolution layers to the reconstruction module, which can be formulated as

(3) $$X_{aggregate}=F_{aggregate}\left(Concat\left(X_{1},\cdots ,X_{n}\right)\right)$$

where Concat represents the concatenation operation, and $F_{aggregate}$ denotes a composite function of a convolution layer with a kernel size of 1×1 following a convolution layer with a kernel size of 3×3.

In addition, the deep-aggregated feature $X_{aggregate}$ is added to the shallow feature $X_{0}$ through global residual learning. Finally, the super-resolving output images are produced through the reconstruction function, as follows

(4) $$I_{SR}=F_{rec}\left(X_{aggregate}+X_{0}\right)$$

where $F_{rec}\left(\cdot \right)$ represents the reconstruction module function and $I_{SR}$ is the output super-resolution image of the network. The reconstruction module consists of a 3×3 convolutional layer and a pixel-shuffle layer.

Different loss functions have been introduced to optimize SR networks. For fair comparison with the most advanced methods, our model is optimized using the $L_{1}$ loss function, as in previous works [18,21]. Given a training set ${\left\{I_{LR}^{i},I_{HR}^{i}\right\}}_{i=1}^{N}$, *N* denotes the number of LR–HR image patches. Hence, the loss function of our AIDN can be represented as

(5) $$L\left(\Theta \right)=\frac{1}{N}\sum_{i=1}^{N}\left|\right|H_{AIDN}\left(I_{LR}^{i}\right)-I_{HR}^{i}{\left|\right|}_{1}$$

where Θ indicates the learnable parameters of our AIDN model and $H_{AIDN}\left(\cdot \right)$ denotes the function of our model. Our goal is to minimize the $L_{1}$ loss function between the reconstructed image $I_{SR}$ and the corresponding ground-truth high-resolution (HR) image $I_{HR}$.

### 3.2. Attention Information Distillation Block

**Figure 2:**
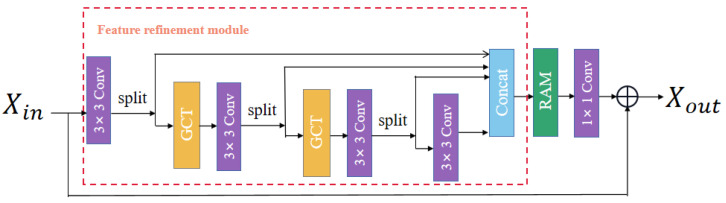
Attention information distillation block (AIDB).

This section mainly introduces the key parts of the proposed AIDB. As shown in Figure 2, the proposed attention information distillation block (AIDB) mainly contains the feature refinement module (FRM) and the recalibrated attention module (RAM). Specifically, the FRM module gradually extracts the multi-layer features by employing information diffluence to obtain a discriminative learning ability. A few features are also aggregated according to their contributions. Moreover, the RAM module re-highlights the informativeness of the features and enhances the expression capability of the network.

### 3.3. Feature Refinement Module

The feature refinement module (FRM) exploits the distillation network and attention mechanism to separate and process features by connection or convolution. Specifically, a 3×3 convolution layer is first exploited to extract input features for multiple succeeding distillation steps in the FRM. For each step, the channel split operation is performed on the previous features, resulting in two-part features. Both parts require further processing. One part is reserved, while the other part is used as input to the gate channel transformation (GCT) module [34]. Assuming the input features are denoted by $X_{in}$, this procedure can be formulated as

(6) $$\begin{array}{c} X_{retain_{1}},X_{coarse_{1}}=Split_{1}\left(F_{conv_{1}}\left(X_{in}\right)\right)\\ X_{retain_{2}},X_{coarse_{2}}=Split_{2}(F_{conv_{2}}\left(F_{GCT_{1}}\left(X_{coarse_{1}}\right)\right)\\ X_{retain_{3}},X_{coarse_{3}}=Split_{3}(F_{conv_{3}}\left(F_{GCT_{2}}\left(X_{coarse_{2}}\right)\right)\\ X_{retain_{4}}=F_{conv_{4}}\left(X_{coarse_{3}}\right) \end{array}$$

where $F_{conv_{i}}$ indicates the *i*-th 3×3 convolution operation followed by the Leaky ReLU(LReLU) activation function, $F_{GCT_{i}}$ denotes the channel transformation operation (detailed in the following section), $Split_{j}$ represents the *j*-th channel split operation, $X_{retain_{i}}$ denotes the *i*-th retained features, and $X_{coarse_{j}}$ represents the *j*-th coarse features, which are further fed to the subsequent layers. Afterward, all the features retained in each step are concatenated along the channel dimension, which can be denoted as

(7) $$X_{FRM}=Concat\left(F_{distilled_{1}},F_{distilled_{2}},F_{distilled_{3}},F_{distilled_{4}}\right)$$

where Concat indicates the concatenation operation and $X_{FRM}$ denotes the output of the feature refinement module (FRM).

### 3.4. Gate Channel Transformation

**Figure 3:**
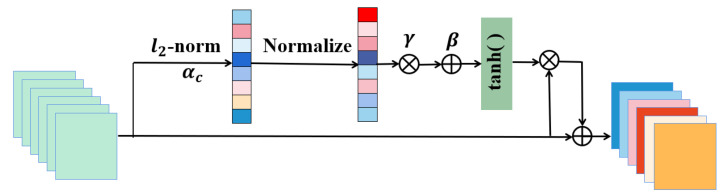
Gate channel transformation module (GCT).

Gate channel transformation (GCT) [34] is an attention mechanism. Moreover, GCT is a simple and effective channel-relationship-modeling architecture, combining a normalization module and gating mechanism. As shown in Figure 3, the overall structure of the GCT module consists three parts: global context embedding, channel normalization, and a gating mechanism. First, we employ $L_{2}$-norm to capture global contextual information from the input feature. Given the input feature $X=\{x_{1},x_{2},...,x_{k}\}$, $X\in R^{C\times H\times W}$, it can be written mathematically as [34]

(8) $$s_{c}=\alpha_{c}\left|\right|x_{c}{\left|\right|}_{2}=\alpha_{c}{\left\{\left[\sum_{i=1}^{H}\sum_{j=1}^{W}{\left(x_{c}^{i,j}\right)}^{2}\right]+\epsilon \right\}}^{\frac{1}{2}}$$

where $S=\{S_{1},S_{2},...,S_{c}\}$, $S\in R^{C\times 1\times 1}$ is the gathered global-context-embedding information along each channel dimension, ε represents a very small constant to avoid the derivation problem at zero point, and $\alpha_{c}$ denotes the trainable parameter, namely the embedding weight. Furthermore, $\alpha_{c}$ can control the different weights of each channel. In particular, when $\alpha_{c}$ approaches 0, the channel will not participate in the subsequent normalization module. Accordingly, it enables the network to recognize when one channel is independent of the others. Then, we adopt the normalization operation to reduce the number of parameters and improve the computational efficiency. Furthermore, normalization approaches [40] have been shown to establish competitive relations between different neurons (or channels) in neural networks, which stabilize the training process. This allows for larger values with larger channel responses and restrains the other channels with less feedback. The channel normalization function can be expressed as

(9) $${\hat{s}}_{c}=\frac{\sqrt{C}s_{c}}{{\left||s|\right|}_{2}}=\frac{\sqrt{C}s_{c}}{{\left[\left(\sum_{c=1}^{C}s_{c}^{2}\right)+\epsilon \right]}^{\frac{1}{2}}}$$

where *C* is the number of channels. Finally, the gating mechanism is introduced to control the activation of the gate channel. The gating function is defined as follows

(10) $${\hat{x}}_{c}=x_{c}\left[1+tanh\left(\gamma_{c}{\hat{s}}_{c}+\beta_{c}\right)\right]$$

where $\gamma =[\gamma_{1},...,\gamma_{C}]$ denotes gating weights, $\beta =[\beta_{1},...,\beta_{C}]$ represents gating biases, and $x_{c}$, ${\hat{x}}_{c}$ are the input and output features of the gating mechanism module, respectively. The weights and biases determine the behavior of GCT in each channel. When the gating weight $\gamma_{C}$ is activated actively, GCT enhances this channel to compete with the others. When the gating weight is activated passively, GCT pushes the channel to cooperate with the others. In other words, low-level features are primarily learned in the shallow layers of the network. Thus, cooperation between channels is required to more widely extract features. In the deeper layers, high-level features are mainly learned, and their differences are often large. Therefore, competition between channels is needed to obtain more valuable feature information.

In addition, when the gating weight and bias are zeros, the original features are allowed to pass to the next layer, which can be formulated as

(11) $${\hat{x}}_{c}=x_{c}$$

This can establish an identity mapping and solve the degradation problem of deep networks. Hence, during GCT module initialization, α is initialized with 1, and γ and β are initialized with 0. The initial steps will be improved the robustness of the training process, and the final GCT results will be more accurate.

### 3.5. Recalibrated Attention Module

**Figure 4:**
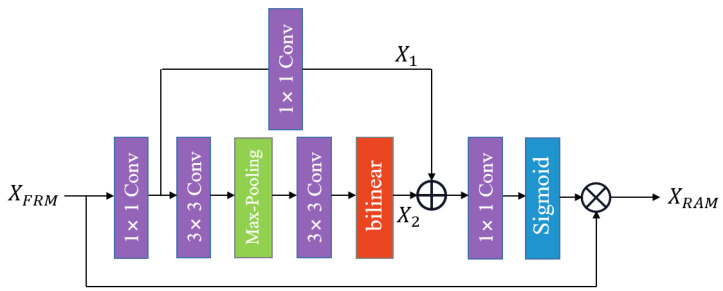
Recalibrated attention module (RAM).

To recalibrate informative features, the output features of FRM are further fed into the recalibrated attention module (RAM), where the informative features are selectively emphasized and useless features are inhibited according to their importance. As shown in Figure 4, the overall structure of the RAM is a bottleneck architecture. Here, $X_{FRM}$ and $X_{RAM}$ are defined as the input and output of the RAM, respectively. Specifically, the concatenated features are first passed to a 1×1 convolution layer to decrease channel dimensions; then, they are divided into two branches. One branch preserves the original information with a 1×1 convolution to produce $X_{1}$, while the other processes the spatial information to search for the areas with the highest contribution. In addition, this branch is equipped with two 3×3 convolutions, a max-pooling layer, and a bilinear interpolation operator to generate $X_{2}$. The max-pooling operation not only enhances the receptive field but also captures high-frequency details. The bilinear interpolation layer maps the intermediate features to the original feature space to keep the identical size of the input and output. Finally, $X_{1}$ and $X_{2}$ are concatenated and fed into a 1×1 convolution followed by a sigmoid function. This 1×1 convolution is adopted to restore the channel dimensions. Hence, the recalibrated attention can be expressed as

(12) $$X_{RAM}=F_{RAM}\left(X_{FRM}\right)\cdot X_{FRM}$$

where $F_{RAM}\left(\cdot \right)$ is the recalibrated attention module function.

Therefore, the final output of the attention information distillation block (AIDB) can be formulated as

(13) $$X_{B^{n}}=X_{B^{n-1}}+F_{conv}\left(X_{RAM}\right)$$

where $F_{conv}$ is a 3×3 convolutional layer, and $X_{B^{n}}$ and $X_{B^{n-1}}$ denote the input and output of the *n*-th AIDB, respectively. Furthermore, the GCT module considers the channel-wise statistics, while the recalibrated attention module (RAM) encodes multi-scale features, focusing on the context around the spatial locations. Therefore, AIDB can modulate more informative features to obtain a more powerful feature representation capability, which is conducive to improving SR performance.

## 4. Experiments Section

In this section, we first describe our experimental conditions regarding the implementation details and training settings. Then, we study the validity of the proposed modules in our model. Finally, we systematically compare the proposed network with plenty of state-of-the-art models.

### 4.1. Datasets and Metrics

In our experiments, following previous works [18,21], we employed the DIV2K dataset [41] to train our model. It includes 800 high-quality training images. In the testing phase, we adopted five public benchmark datasets—Set5 [42], Set14 [43], BSD100 [44], Urban100 [45], and Manga109 [46]—to comprehensively validate the effectiveness of our model. In addition, we leveraged the peak signal-to-noise ratio (PSNR) and the structural similarity index (SSIM) [47] as quantitative evaluation metrics for the performance of the final reconstructed super-resolution images. We computed the PSNR and SSIM values on the luminance channel of the YCbCr color space. We also compared parameter amounts with other leading models. Given a ground-truth image $I^{HR}$ and a super-resolved image $I^{SR}$, we defined the PSNR as:

(14) $$PSNR\left(I^{HR},I^{SR}\right)=10log_{10}\left(\frac{Max_{I}^{2}}{MSE}\right)$$

where

(15) $$MSE=\frac{1}{H\times W}\sum_{i=1}^{H}\sum_{j=1}^{W}{\left(I^{HR}\left(i,j\right)-I^{SR}\left(i,j\right)\right)}^{2}$$

$Max_{I}$ is the maximum pixel value of an image, and *H* and *W* are the height and width, respectively. We formulated SSIM as:

(16) $$SSIM\left(I^{HR},I^{SR}\right)=l\left(I^{HR},I^{SR}\right)c\left(I^{HR},I^{SR}\right)s\left(I^{HR},I^{SR}\right)$$

where

(17) $$\left\{\begin{array}{cc} l\left(I^{HR},I^{SR}\right)=\frac{2\mu_{I^{HR}}\mu_{I^{SR}}+C_{1}}{\mu_{I^{HR}}^{2}+\mu_{I^{SR}}^{2}+C_{1}} & \\ c\left(I^{HR},I^{SR}\right)=\frac{2\sigma_{I^{HR}}\sigma_{I^{SR}}+C_{2}}{\sigma_{I^{HR}}^{2}+\sigma_{I^{SR}}^{2}+C_{2}} & \\ s\left(I^{HR},I^{SR}\right)=\frac{2\sigma_{I^{HR}I^{SR}}+C_{3}}{\sigma_{I^{HR}}\sigma_{I^{SR}}+C_{3}} \end{array}\right.$$

$\mu_{I^{HR}}$, $\sigma_{I^{HR}}$, and $\sigma_{I^{HR}I^{SR}}$ are the mean, standard deviation, and covariance of an image, respectively, and $C_{1}$, $C_{2}$, and $C_{3}$ are positive constants.

### 4.2. Implementation Details

#### 4.2.1. Training Settings

We obtained the input low-resolution (LR) images from the corresponding HR images by bicubic down-sampling in the training stage. Then, we set 16 LR patches as each training mini-batch, and extracted with a size of 48×48 from the LR images. Moreover, we randomly rotated the image in the training dataset by $90^{\circ }$, $180^{\circ }$, and $270^{\circ }$, and flipped it horizontally for data augmentation. We utilized Adam optimizer [48] to optimize our model with settings of $\beta_{1}=0.9$ and $\beta_{2}=0.999$. We fixed the initial learning rate to $2\times 10^{-4}$, and decreased by half every 200 epochs. We performed the proposed model on the PyTorch framework with an NVIDIA GTX 1080Ti GPU. More setting details of our experiments are listed in Table 1.

#### 4.2.2. Model Details

Our model includes six attention information distillation blocks (AIDBs), and we set the number of feature channels to 64. Among them, we reserved the channels=16 and further processed the other parts. We set the activation functions in the feature refinement module (FRM) as LReLU, while we applied ReLU to the other parts [49]. Additionally, in the recalibrated attention module (RAM), we deployed the first 3×3 convolution layer with a stride = 2, the other 3×3 convolution layer with stride = 1, and used the max-pooling operation with a 7×7 convolution with stride = 3.

### 4.3. Study of GCT and RAM

To study the contributions of the different modules in the proposed model, we conducted ablation experiments. All the models are trained from scratch for 1000 epochs, and are executed under similar settings. Each time we removed one module, we directly tested the model performance without adding other operations. Table 2 shows the experimental results at a scale factor of 2 on multiple datasets. Without gate channel transformation (GCT) and the recalibrated attention module (RAM) in the information distillation block (AIDB), the PSNR values of all datasets are relatively lower. The performance of the second row with GCT module is better than that of the first row with only 1 K more parameters. Similarly, RAM in the third row also improves the performance, especially on Urban100 and Manga109 datasets. Therefore, both the GCT module and RAM can independently obtain better reconstruction accuracy. This can be attributed to the multi-layer features being discriminatively treated, and different weights being allocated according to the characteristics of features to screen out high-value information features, improving the efficiency and accuracy of the network. Furthermore, the best reconstruction results are provided when integrating GCT and RAM into the AIDB with few additional parameters, as shown in the last row of Table 2. Thus, the proposed AIDB can capture spatial and global contextual information in each channel, benefiting image restoration. The above quantitative results effectively prove the effectiveness of the network structure with the introduced GCT and RAM, and their integration.

### 4.4. Comparison with State-of-the-Art Methods

To demonstrate the effectiveness of our proposed architecture, we compared recently proposed competitive works, including SRCNN [5], VDSR [6], DRCN [7], LapSRN [20], IDN [18], CARN [26], MoreMNAS-A [50], FALSR-A [29], ESRN-V [15], WMRN [21], MADNet-$L_{1}$ [19], MSICF [51], and CFSRCNN [22], with the proposed network. These works are almost all lightweight networks with less than 2.0M parameters. The quantitative results with scale factors of ×2, ×3, and ×4 on five benchmark datasets are provided in Table 3. It can be seen that our proposed model is superior to the other leading algorithms across different datasets and scaling factors. Specifically, compared with several automatic search SR architectures based on NAS (FALSR-A, MoreMNAS, and ESRN-V), our AIDN network gets higher PSNR values with fewer parameters (FLOPs) on five datasets for ×2 up-scaling.

Although WMRN has slightly fewer parameters than the proposed network, its reconstruction results are far worse. For example, with a scale factor of 3 on Set5, our network obtains a significant performance gain of 0.24 dB. Moreover, our AIDN performs well compared to MADNet-$L_{1}$, which has a similar number of parameters. MADNet also applied an attention mechanism with a residual multi-scale module. For a scale factor of 4 on four datasets, CFSRCNN achieves the second-best performance with nearly twice as many parameters as our method. From Table 3, it can be seen that the number of parameters of SRCNN and VDSR do not change across scaling factors, as the input image is interpolated and then sent into the network. Other models have varying parameters due to different up-sampling approaches. As our model has relatively few parameters, it can be considered a lightweight model. Consequently, our method has better reconstruction performance than the most advanced methods, with fewer parameters.

**Figure 5:**
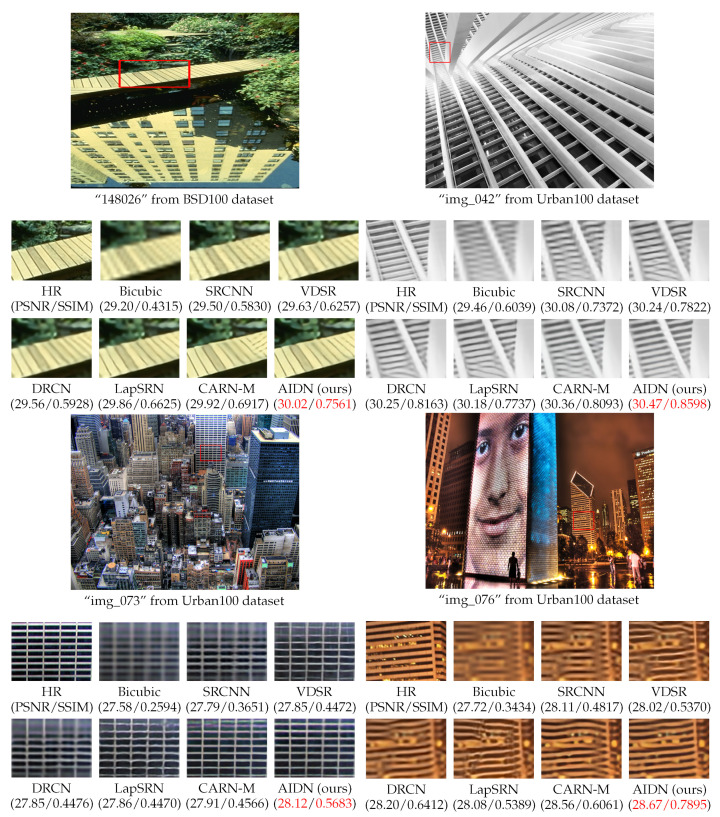
Visual comparison results of our AIDN with SRCNN [5], VDSR [6], DRCN [7], LapSRN [20] and CARN-M [26] for ×4 SR images on BSD100 and Urban100 dataset. The best results are highlighted by red.

In addition, we also compared the visual quality with other methods at the ×4 scale, as shown in Figure 5. For “148026” from BSD100, most methods restored the blurred edges, while CARN even produced wrong textures; furthermore, the image generated by our method was closer to the original image. For “img_042” from Urban100, other methods suffered from severe artifacts, and the lines produced are curved. Only the image refined by our method outputted horizontal lines correctly. For “img_037” from Urban100, LapSRN could not recover grids, and VDSR rebuilt several redundant white vertical lines at the upper right of the image. Our AIDN reconstructed accurate grids with better visual effects.

### 4.5. Heatmaps of the Proposed AIDN

**Figure 6:**
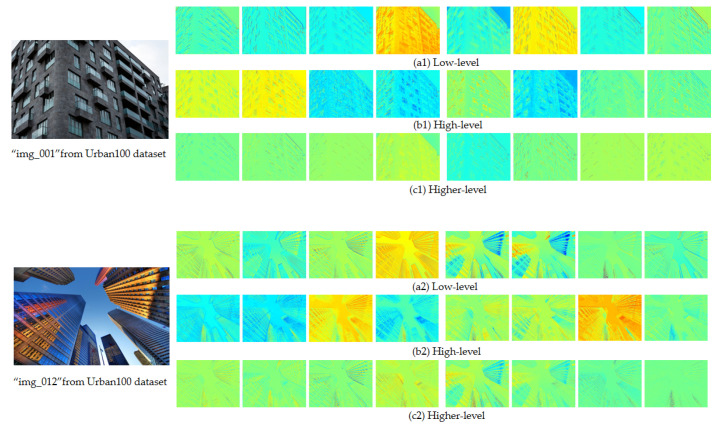
Heatmaps of our AIDN at different stages from Urban100 dataset.

This section describes the heatmaps of the proposed AIDN at stages with the Urban100 dataset (×2). In Figure 6, the top row shows heatmaps of the shallow features before passing into AIDBs, while the next two rows are heatmaps of the refined high-level features. The results show that our method has different weights in different states. The rows represent the states of different channels at the same time, and the columns represent the states of the same channel at different times. Yellow is heavily weighted, blue is lightly weighted, and green is centered. It can be seen that our method has the function of modulating features, which is conducive to image reconstruction.

### 4.6. Model Size Analysis

**Figure 7:**
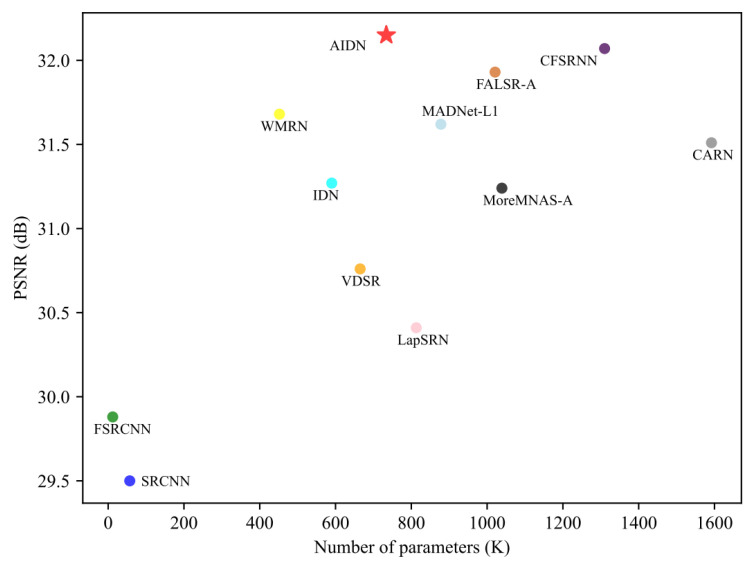
Our AIDN compared with other models in terms of parameters and performance. Results are evaluated on Urban100 with a scale factor of 2.

In addition, to further illustrate the superiority of the proposed network, we compared its number of parameters and performance with other leading works. The number of parameters is especially important when building a lightweight network, especially for resource-constrained mobile devices. The experimental results on Urban100 with a scale factor of ×2 are shown in Figure 7. Compared with other methods, our AIDN model obtained comparable or higher PSNR values with fewer parameters, while other methods either had a larger number of parameters or lower performance. These analyses indicate that the proposed AIDN strikes a better balance between parameters and performance.

### 4.7. Visualization on Historical Images

**Figure 8:**
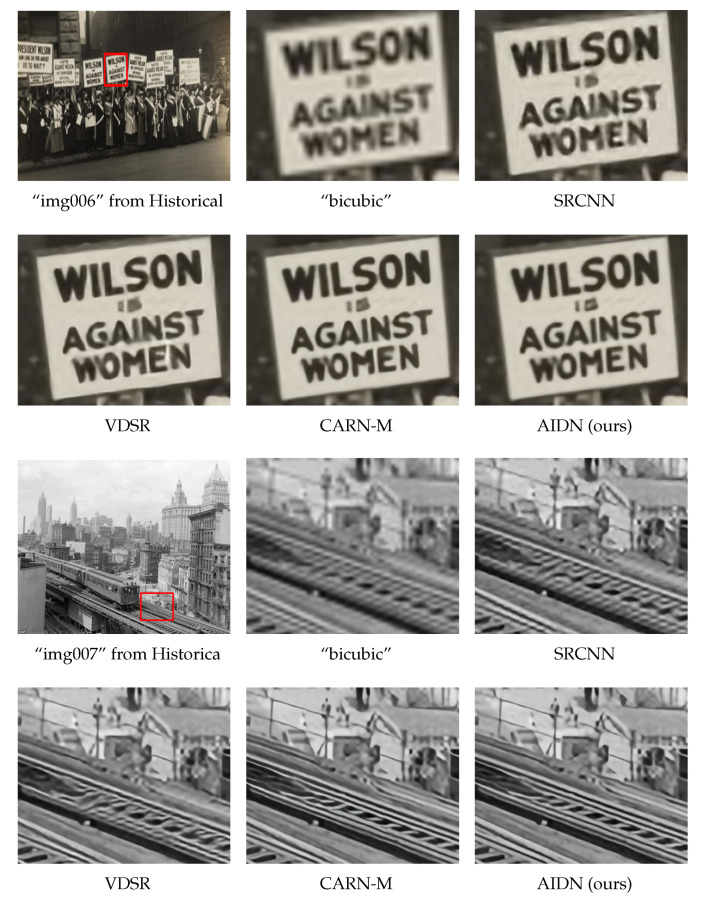
Visual comparisons for a scale factor of ×4 SR on historical images.

To further illustrate the robustness and effect of our model, we evaluated our attention network with information distillation (AIDN) on historical images. The degradation process of these low-resolution images is unknown, and no corresponding high-quality images are available. Figure 8 shows the visual results on scale factor ×4. For “img006”, the characters produced by our model were clearer and more independent. For “img007”, our AIDN could reconstruct finer details, and the refined images showed lower blurring. In short, the images generated by our method have better perceptual quality than those of other methods.

## 5. Conclusions

In this paper, we proposed an attention network with information distillation (AIDN) for image super-resolution. Specifically, global contextual information embedding among different channels is employed to modulate multiple features in a step-by-step manner, forming the distillation structure. Moreover, a recalibrated attention module (RAM) is adopted to re-highlight these features, concentrating on the vital contents around spatial locations. Benefiting from the gated channel transformation and spatial information unit masks working jointly, the proposed AIDN possesses a more powerful information identifying capability, effectively improving the computational efficiency while enhancing the reconstruction accuracy. Comprehensive quantitative and qualitative evaluations effectively demonstrate that our AIDN outperforms state-of-the-art models in terms of both reconstruction performance and visual quality. In future work, we will extend our AIDN to other complex tasks (e.g., images with noise, blurring, etc.).

## Figures and Tables

**Table 1:** Setting parameters for our AIDN.

Batch size	48 × 48
Patch size	16
The numbers of information distillation blocks	6
Initial learning rate	$2\times 10^{-4}$
Channels	64
Channels—reserved (split)	16
Optimizer (Adam)	$\beta_{1}=0.9$, $\beta_{2}=0.999$

**Table 2:** Investigations of GCT module and RAM unit on five benchmark datasets at scaling factors of ×2. PSNR/SSIM represent the two values. Params: kernel*kernel*channel-input*channel-output. The best and second-best performances are highlighted in red and blue.

Scale	GCT	RAM	Params	Set5	Set14	BSD100	Urban100	Manga109
			(K)	PSNR/SSIM	PSNR/SSIM	PSNR/SSIM	PSNR/SSIM	PSNR/SSIM
×2	×	×	690	37.63/0.9584	31.30/0.9146	31.94/0.8966	31.31/0.9199	37.79/0.9744
×2	✔	×	691	37.81/0.9591	33.41/0.9157	32.07/0.8980	31.75/0.9241	38.27/0.9754
×2	×	✔	733	37.85/0.9592	33.51/0.9167	32.10/0.8982	31.94/0.9260	38.44/0.9758
×2	✔	✔	734	37.95/0.9596	33.57/0.9169	32.16/0.8989	32.16/0.9278	38.68/0.9763

**Table 3:** Quantitative results of several state-of-the-art SR models at scaling factors of ×2, ×3 and ×4 (average PSNR/SSIM). The best performance is highlighted in red, while the second-best performance is highlighted in blue.

Method	Scale	Params	Set5	Set10	BSD100	Urban100	Manga109
		(K)	PSNR/SSIM	PSNR/SSIM	PSNR/SSIM	PSNR/SSIM	PSNR/SSIM
SRCNN [5]	×2	57	36.66/0.9542	32.42/0.9063	31.36/0.8879	29.50/0.8946	35.60/0.9663
VDSR [6]	×2	665	37.53/0.9587	33.03/0.9124	31.90/0.8960	30.76/0.9140	37.22/0.9729
LapSRN [20]	×2	813	37.52/0.9590	33.08/0.9130	31.80/0.8950	30.41/0.9100	37.27/0.9740
IDN [18]	×2	590	37.83/0.9600	33.30/0.9148	32.08/0.8950	31.27/0.9196	-
CARN-M [26]	×2	412	37.53/0.9583	33.26/0.9141	31.92/0.8960	30.83/0.9233	-
AWSRN-S [12]	×2	397	37.75/0.9596	33.31/0.9151	32.00/0.8974	31.39/0.9207	37.90/0.9755
ESRN-V [15]	×2	324	37.85/0.9600	33.42/0.9161	32.10/0.8987	31.79/0.9248	-
WMRN [21]	×2	452	37.83/0.9599	33.41/0.9162	32.08/0.8984	31.68/0.9241	38.27/0.9763
MADNet-L1 [19]	×2	878	37.85/0.9600	33.38/0.9161	32.04/0.8979	31.62/0.9233	-
MoreMNAS-A [50]	×2	1039	37.63/0.9584	33.23/0.9138	31.95/0.8961	31.24/0.9187	-
MSICF [51]	×2	1900	37.89/0.9605	33.41/0.9153	32.15/0.8992	31.47/0.9220	-
FALSR-A [29]	×2	1021	37.82/0.9595	33.55/0.9168	32.12/0.8987	31.93/0.9256	-
CFSRCNN [22]	×2	1310	37.79/0.9591	33.51/0.9165	32.11/0.8988	32.07/0.9273	-
AIDN(ours)	×2	734	37.95/0.9603	33.57/0.9169	32.16/0.8993	32.16/0.9278	38.68/0.9782
SRCNN [5]	×3	57	32.75/0.9090	29.28/0.8209	28.41/0.7863	26.24/0.7989	30.59/0.9107
VDSR [6]	×3	665	33.66/0.9213	29.77/0.8314	28.82/0.7976	27.14/0.8279	32.01/0.9310
IDN [18]	×3	590	34.11/0.9253	29.99/0.8354	28.95/0.8013	27.42/0.8359	-
CARN-M [26]	×3	412	33.99/0.9236	30.08/0.8367	28.91/0.8000	26.86/0.8263	-
AWSRN-S [12]	×3	447	34.02/0.9240	30.09/0.8376	28.92/0.8009	27.57/0.8391	32.82/0.9393
ESRN-V [15]	×3	324	34.23/0.9262	30.27/0.8400	29.03/0.8039	27.95/0.8481	-
WMRN [21]	×3	556	34.11/0.9251	30.17/0.8390	28.98/0.8021	27.80/0.8448	33.07/0.9413
MADNet-L1 [19]	×3	930	34.16/0.9253	30.21/0.8398	28.98/0.8023	27.77/0.8439	-
MSICF [51]	×3	1900	34.24/0.9266	30.09/0.8371	29.01/0.8024	27.69/0.8411	-
CFSRCNN [22]	×3	1495	34.24/0.9256	30.27/0.8410	29.03/0.8035	28.04/0.8496	-
AIDN(ours)	×3	742	34.35/0.9259	30.35/0.8413	29.07/0.8039	28.13/0.8512	33.50/0.9433
SRCNN [5]	×4	57	30.48/0.8628	27.49/0.7503	26.90/0.7101	24.52/0.7221	27.66/0.8505
VDSR [6]	×4	665	31.35/0.8838	28.01/0.7674	27.29/0.7251	25.18/0.7524	28.83/0.8809
LapSRN [20]	×4	813	31.54/0.8850	28.19/0.7720	27.32/0.7280	25.21/0.7560	29.09/0.8845
IDN [18]	×4	590	31.82/0.8903	28.25/0.7730	27.41/0.7297	25.41/0.7632	-
CARN-M [26]	×4	412	31.92/0.8903	28.42/0.7762	27.44/0.7304	25.63/0.7688	-
AWSRN-S [12]	×4	588	31.77/0.8893	28.35/0.7761	27.41/0.7304	25.56/0.7678	29.74/0.8982
ESRN-V [15]	×4	324	31.99/0.8919	28.49/0.7779	27.50/0.7331	25.87/0.7782	-
WMRN [21]	×4	536	32.00/0.8925	28.47/0.7786	27.49/0.7328	25.89/0.7789	30.11/0.9040
MADNet-L1 [19]	×4	1002	31.95/0.8917	28.44/0.7780	27.47/0.7327	25.76/0.7746	-
MSICF [51]	×4	1900	31.91/0.8923	28.35/0.7751	27.46/0.7308	25.64/0.7692	-
CFSRCNN [22]	×4	1458	32.06/0.8920	28.57/0.7800	27.53/0.7333	26.03/0.7824	-
AIDN(ours)	×4	754	32.17/0.8939	28.61/0.7806	27.55/0.7344	26.04/0.7833	30.42/0.9065

## Data Availability

Not applicable.

## References

[1] Russakovsky O., Deng J., Su H., Krause J., Satheesh S., Ma S., Huang Z., Karpathy A., Khosla A., Bernstein M. (2015). ImageNet Large Scale Visual Recognition Challenge. Int. J. Comput. Vis..

[2] Guo T., Dai T., Liu L., Zhu Z., Xia S.-T. (2021). S2A:Scale Attention-Aware Networks for Video Super-Resolution. Entropy.

[3] Tang R., Chen L., Zou Y., Lai Z., Albertini M.K., Yang X. (2021). Lightweight network with one-shot aggregation for image super-resolution. J. Real-Time Image Process..

[4] Jiang X., Wang N., Xin J., Xia X., Yang X., Gao X. (2021). Learning lightweight super-resolution networks with weight pruning. Neural Netw..

[5] Dong C., Loy C.C., He K., Tang X. Learning a deep convolutional network for image super-resolution. Proceedings of the European Conference on Computer Vision—ECCV 2014.

[6] Kim J., Lee J.K., Lee K.M. Accurate image super-resolution using very deep convolutional networks. Proceedings of the 2016 IEEE Conference on Computer Vision and Pattern Recognition (CVPR).

[7] Kim J., Lee J.K., Lee K.M. Deeply-recursive convolutional network for image super-resolution. Proceedings of the 2016 IEEE Conference on Computer Vision and Pattern Recognition (CVPR).

[8] He K., Zhang X., Ren S., Sun J. Deep residual learning for image recognition. Proceedings of the 2016 IEEE Conference on Computer Vision and Pattern Recognition (CVPR).

[9] Dong C., Chen C.L., Tang X.O. Accelerating the Super-Resolution Convolutional Neural Network. Proceedings of the 2016 European Conference on Computer Vision (ECCV).

[10] Shi W., Caballero J., Huszár F., Totz J., Aitken A.P., Bishop R., Rueckert D., Wang Z. Real-time single image and video super-resolution using an efficient sub-pixel convolutional neural network. Proceedings of the 2016 IEEE Conference on Computer Vision and Pattern Recognition (CVPR).

[11] Lim B., Son S., Kim H., Nah S., Lee K.M. Enhanced deep residual networks for single image super-resolution. Proceedings of the 2017 IEEE Conference on Computer Vision and Pattern Recognition Workshops (CVPRW).

[12] Li Z., Wang C., Wang J., Ying S., Shi J. (2021). Lightweight adaptive weighted network for single image super-resolution. Comput. Vis. Image Underst..

[13] Tong T., Li G., Liu X.J., Gao Q.Q. Image Super-Resolution Using Dense Skip Connections. Proceedings of the 2017 IEEE/CVF International Conference on Computer Vision (ICCV).

[14] Huang G., Liu Z., Maaten L.V.D., Weinberger K.Q. Densely connected convolutional networks. Proceedings of the 2017 IEEE Conference on Computer Vision and Pattern Recognition (CVPR).

[15] Song D., Xu C., Jia X., Chen Y., Xu C., Wang Y. Efficient residual dense block search for image super-resolution. Proceedings of the Thirty-Fourth AAAI Conference on Artificial Intelligence (AAAI).

[16] Zoph B., Le Q.V. Neural architecture search with reinforcement learning. Proceedings of the International Conference on Learning Representations.

[17] Zhang Y.L., Li K.P., Li K., Wang L.C., Zhong B.E., Fu Y. Image Super-Resolution Using Very Deep Residual Channel Attention Networks. Proceedings of the 2018 European Conference on Computer Vision (ECCV).

[18] Hui Z., Wang X., Gao X. Fast and accurate single image superresolution via information distillation network. Proceedings of the 2018 IEEE Conference on Computer Vision and Pattern Recognition (CVPR).

[19] Lan R., Sun L., Liu Z., Lu H., Pang C., Luo X. (2021). MADNet: A Fast and Lightweight Network for Single-Image Super Resolution. IEEE Trans. Cybern..

[20] Lai W.S., Huang J.B., Ahuja N., Yang M.H. Deep Laplacian pyramid networks for fast and accurate super-resolution. Proceedings of the 2017 IEEE Conference on Computer Vision and Pattern Recognition (CVPR).

[21] Sun L., Liu Z., Sun X., Liu L., Lan R., Luo X. (2021). Lightweight Image Super-Resolution via Weighted Multi-Scale Residual Network. IEEE/CAA J. Autom. Sin..

[22] Tian C., Xu Y., Zuo W., Zhang B., Fei L., Lin C.W. (2020). Coarse-to-fine cnn for image super-resolution. IEEE Trans. Multimed..

[23] Liu J., Zhang W.J., Tang Y.T., Tang J., Wu G.S. Residual Feature Aggregation Network for Image Super-Resolution. In Proceedings of the 2020 IEEE Conference on Computer Vision and Pattern Recognition (CVPR).

[24] Zhang Y., Tian Y., Kong Y., Zhong B., Fu Y. Residual dense network for image super-resolution. Proceedings of the 2018 IEEE Conference on Computer Vision and Pattern Recognition (CVPR).

[25] Zhang X.Y., Gao P., Liu S.X., Zhao K.Y., Yin L.G., Chen C.W. (2021). Accurate and Efficient Image Super-Resolution via Global-Local Adjusting Dense Network. IEEE Trans. Multimed..

[26] Ahn N., Kang B., Sohn K. Fast, accurate, and lightweight super-resolution with cascading residual network. Proceedings of the 2018 European Conference on Computer Vision (ECCV).

[27] Zhu F.Y., Zhao Q.J. Efficient single image super-resolution via hybrid residual feature learning with compact back-projection network. Proceedings of the 2019 IEEE/CVF International Conference on Computer Vision Workshop (ICCVW).

[28] Song D.H., Wang Y.H., Chen H.T., Xu C.J., Tao D.C. AdderSR: Towards Energy Efficient Image Super-Resolution. Proceedings of the 2021 IEEE Conference on Computer Vision and Pattern Recognition (CVPR).

[29] Chu X., Zhang B., Ma H., Xu R., Li J., Li Q. Fast, accurate and lightweight super-resolution with neural architecture search. Proceedings of the Conference: 2020 25th International Conference on Pattern Recognition (ICPR).

[30] Chen G.A., Matsune A., Du H., Liu X.Z., Zhan S. (2022). Exploring more diverse network architectures for single image super-resolution. Knowl.-Based Syst..

[31] Hu J., Shen L., Sun G. Squeeze-and-Excitation Networks. Proceedings of the 2018 IEEE Conference on Computer Vision and Pattern Recognition (CVPR).

[32] Wang X.L., Girshick R., Gupta A., He K.M. Non-local Neural Networks. Proceedings of the 2018 IEEE Conference on Computer Vision and Pattern Recognition (CVPR).

[33] Woo S.H.Y., Park J.C., Lee J.Y., Kweon I.S. CBAM: Convolutional Block Attention Module. Proceedings of the European Conference on Computer Vision (ECCV).

[34] Yang Z.X., Zhu L.C., Wu Y., Yang Y. Gated Channel Transformation for Visual Recognition. Proceedings of the 2020 IEEE Conference on Computer Vision and Pattern Recognition (CVPR).

[35] Wang Q.L., Wu B.G., Zhu P.F., Li P.H., Zuo W.M., Hu Q.H. ECA-Net: Efficient Channel Attention for Deep Convolutional Neural Networks. Proceedings of the 2020 IEEE Conference on Computer Vision and Pattern Recognition (CVPR).

[36] Dai T., Cai J.R., Zhang Y.B., Xia S.T., Zhang L. Second-order Attention Network for Single Image Super-Resolution. Proceedings of the 2019 IEEE Conference on Computer Vision and Pattern Recognition (CVPR).

[37] Anwar S., Barnes N. (2020). Densely Residual Laplacian Super-Resolution. IEEE Trans. Pattern Anal. Mach. Intell..

[38] Hu Y.T., Li J., Huang Y.F., Gao X.B. (2020). Channel-wise and spatial feature modulation network for single image super-resolution. IEEE Trans. Circuits Syst. Video Technol..

[39] Mei Y.Q., Fan Y.C., Zhou Y.Q., Huang L.C. Image Super-Resolution with Cross-Scale Non-Local Attention and Exhaustive Self-Exemplars Mining. Proceedings of the 2020 IEEE Conference on Computer Vision and Pattern Recognition (CVPR).

[40] Krizhevsky A., Sutskever I., Hinton G. (2012). ImageNet Classification with Deep Convolutional Neural Networks. Adv. Neural Inf. Process. Syst..

[41] Agustsson E., Timofte R. Ntire 2017 challenge on single image super-resolution: Dataset and study. Proceedings of the IEEE Conference on Computer Vision and Pattern Recognition (CVPR) Workshops.

[42] Bevilacqua M., Roumy A., Guillemot C., Morel M.l. Low-complexity single-image super-resolution based on nonnegative neighbor embedding. Proceedings of the British Machine Vision Conference.

[43] Zeyde R., Elad M., Protter M., Boissonnat J.-D., Chenin P., Cohen A., Christian G., Lyche T., Mazure M.-L., Schumaker L. (2012). On single image scale-up using sparse-representations. Curves and Surfaces.

[44] Martin D., Fowlkes C., Tal D., Malik J. A database of human segmented natural images and its application to evaluating segmentation algorithms and measuring ecological statistics. Proceedings of the 8th International Conference on Computer Vision.

[45] Huang J., Singh A., Ahuja N. Single image super-resolution from transformed self-exemplars. Proceedings of the 2015 IEEE Conference on Computer Vision and Pattern Recognition (CVPR).

[46] Matsui Y., Ito K., Aramaki Y., Fujimoto A., Ogawa T., Yamasaki T., Aizawa K. (2017). Sketch-based manga retrieval using manga109 dataset. Multimed. Tools Appl..

[47] Wang Z., Bovik A.C., Sheikh H.R., Simoncelli E.P. (2004). Image quality assessment: From error visibility to structural similarity. IEEE Trans. Image Process..

[48] Kingma D., Ba J. Adam: A method for stochastic optimization. Proceedings of the International Conference on Learning Representations.

[49] Maas A.L., Hannun A.Y., Ng A.Y. Rectifier nonlinearities improve neural network acoustic models. Proceedings of the ICML Workshop on Deep Learning for Audio, Speech and Language Processing.

[50] Chu X., Zhang B., Xu R., Ma H. Multi-objective reinforced evolution in mobile neural architecture search. Proceedings of the European Conference on Computer Vision (ECCV).

[51] Hu Y.T., Gao X.B., Li J., Wang H.Z. (2021). Single image super-resolution with multi-scale information cross-fusion network. Image Process..

